# Does unilateral reflux have a protective effect in posterior urethral valve patients?

**DOI:** 10.1007/s00383-025-06024-8

**Published:** 2025-04-16

**Authors:** Kadir Emre Baltaci, Ali Cansu Bozaci, Mesut Altan, Gökhan Karakurt, Kamranbay Gasimov, Berk Hazir, Serdar Tekgul, Hasan Serkan Dogan

**Affiliations:** 1 Department of Urology, Hacettepe University School of Medicine, Ankara, Turkey; 2 Divison of Pediatric Urology, Department of Urology, Hacettepe University School of Medicine, Ankara, Turkey

**Keywords:** Chronic kidney disease, Pediatric, Pop-off mechanisms, Posterior urethral valve, Vesicoureteral reflux, VURD syndrome

## Abstract

**Objectives:**

To evaluate the protective role of unilateral reflux on renal function in PUV patients. Various protective mechanisms have been proposed in patients with posterior urethral valves (PUV). The role of unilateral reflux as a protective factor is debated.

**Methods:**

A retrospective analysis was conducted on 92 male PUV patients treated between January 1986 and July 2023. Unilateral VUR patients and ipsilateral renal function < 15% were classified as having valve unilateral reflux dysplasia (VURD) syndrome. Data from 92 patients were analyzed using scintigraphy. Renal function was considered abnormal if serum creatinine levels exceeded age-specific norms. Univariate and multivariate analyses assessed various parameters.

**Results:**

The median age at primary surgery was 5 months, with a median follow-up of 35 months. Bilateral VUR patients (55.2%, 16/29) had worse renal outcomes than those with no VUR (25%, 8/32) or unilateral VUR (19.3%, 6/31; (*p* = 0.016, *p* = 0.004, respectively). Thirteen of 31 patients with unilateral VUR had VURD. Abnormal renal function distribution was similar between unilateral VUR patients, regardless of VURD status. Nadir creatinine value were independent risk factors for abnormal renal function.

**Conclusion:**

Although some studies suggest unilateral reflux protects the contralateral kidney, others indicate worse outcomes due to dysplasia. Our findings show that renal outcomes in unilateral VUR patients are comparable with those without VUR, implying a protective effect independent of ipsilateral dysplasia. Patients with unilateral VUR exhibit similar outcomes to those without VUR. Nephrectomy should be cautiously considered due to potential protective effects.

## Introduction

Posterior urethral valve (PUV) is a congenital urethral anomaly affecting male patients, initially identified by Young [1]. This condition impairs both bladder development and the upper urinary system. Given that approximately 20–50% of patients develop chronic kidney disease later in life, with about 40% requiring renal replacement therapy due to end-stage kidney disease, PUV poses a significant burden on the healthcare system [2, 3].

Factors influencing final renal function include the patient’s age, prenatal diagnosis, initial renal function, presence of vesicoureteral reflux (VUR), hydronephrosis status, and bladder function [4–6]. The impact of unilateral high-grade VUR and ipsilateral renal dysplasia (valve unilateral reflux dysplasia [VURD] syndrome), among other factors like urinary ascites, persistent urachus, large bladder diverticulum, and urinoma, on long-term renal function in PUV patients remains controversial [7, 8]. Literature often reflects varying results due to differences among large-scale centers.

Bladder dysfunction leads to the development of vesicoureteral reflux (VUR), obstructive uropathy, and renal dysplasia, collectively known as valve bladder syndrome. Throughout this process, structural changes occur in the bladder [9]. Even after the obstruction is relieved, these structural changes persist, resulting in ongoing bladder dysfunction, a condition referred to as valve bladder [10].

This study aims to evaluate potential prognostic factors for long-term renal function, hypothesizing that unilateral reflux may have a protective effect.

## Material and methods

This retrospective study included 92 male patients who underwent PUV surgery between 1986 and 2023 at a single tertiary pediatric center. DMSA and/or MAG3 scan results were available all patients. Since it is objective data, DMSA or MAG-3, whichever is available, was used. Clinical presentations such as prenatal diagnosis, postnatal urinary symptoms, urinary tract infection, or urosepsis were reviewed. Ages at surgery were categorized as 0–1 year, 1–5 year, and over 5 year. Follow-up durations and final ages were assessed. Patients with serum creatinine levels exceeding age-specific reference values were considered to have abnormal renal function (Table 1) [11]. Patient results were classified into normal and abnormal renal function and analyzed accordingly. The nadir creatinine value, defined as the lowest serum creatinine post-diagnosis and obstruction removal, and 1 year creatinine values were evaluated. The nadir creatinine values of 82 patients, 1 year creatinine values of 41 patients (of 63 patients diagnosed under 1 year. of age), and final creatinine values for all patients were analyzed. All potentially relevant variables were evaluated based on the patients’ final serum creatinine levels exceeding age-specific reference values, and renal function was assessed according to their final status.

**Table 1:** Age-specific reference values

Male	
Age	Serum creatinine (mg/dL)
1–30 days	0.5–1.2
31–365 days	0.4–0.7
1–3 year	0.4–0.7
4–6 year	0.5–0.8
7–9 year	0.6–0.9
10–12 year	0.6–1.0
13–15 year	0.6–1.2
16–18 year	0.8–1.4

Initial treatments included valve ablation under general anesthesia for 75 patients, and vesicostomy/ureterocutaneostomy for 17 patients. The diversion method was performed in patients who presented with severe hydronephrosis or significantly impaired renal function. Additional procedures, such as nephrectomy, augmentation, and transplantation, were also reviewed.

Vesicoureteral reflux patients were categorized as no VUR, unilateral VUR or bilateral VUR. These three groups were analyzed separately. VURD was defined as having a normal contralateral kidney, unilateral VUR, and ipsilateral separate renal function < 15% [12]. Patients with unilateral reflux, with and without VURD, were compared with each other.

Data analysis was performed using SPSS 24.0 (IBM Corp., Chicago). The chi-square test was used for nominal data in univariate analysis, with the median ± range used for nonparametric data. ROC curve analysis determined cut-off values for sensitivity and specificity. Statistically significant factors from univariate analysis were further examined using multivariate logistic regression with a backward stepwise model. A *p* value of < 0.05 was considered statistically significant.

## Results

The median age of the patients was 5 (1–120) months, and the median follow-up duration was 35 (6–300) months. At the last visit, 31 (33.7%) patients had abnormal renal function. Descriptive data and factors affecting renal function are detailed in Table 2.

**Table 2:** Effects of evaluated parameters on renal function (RF) at last visit

			Final RF (age-specific reference values)		Total	*p*
			Normal	Abnormal		
Age at diagnosis (year)	< 1		46	17	63	0.058
	≥ 1		15	14	29	
	< 5		55	23	78	0.064
	≥ 5		6	8	14	
Clinical presentation	Urosepsis		5	8	13	0.029*
	Other	Prenatal diagnosis	25	7	32	
		Voiding disturbances and/or UTI	31	17	48	
Preoperative VUR	Bilateral		13	16	29	0.003*
	Other	Unilateral	25	6	31	
		No VUR	24	8	32	
Nadir creatinine at diagnosis	Normal		38	3	41	< 0.0001*
	High		15	26	41	
1 year creatinine	Normal		29	4	36	< 0.007*
	High		3	5	8	

*p*: Chi-square test

Values with asterisk indicate statistical significance

There was no statistically significant difference in renal function between patients diagnosed before and after 1 or 5 year. of age (Table 2) or between prenatal and postnatal diagnoses (*p* = 0.08). The rate of prenatal diagnosis increased significantly after 2000 (5.3% pre-2000 vs. 42.5% post-2000, *p* = 0.002). Urosepsis was associated with significantly abnormal renal function (*p* = 0.029).

The incidence of abnormal renal function was 42.6% (23/54) for patients followed for over 2 and 51.7% (15/29) for those followed for over 5 year.

In five patients (6.6%) who initially underwent primary valve ablation, secondary diversion was performed—three with vesicostomy and two with ureterocutaneostomy—due to residual valves detected in follow-up cystoscopies. All of these patients exhibited good renal function at the final evaluation.

For patients diagnosed under 1 year. of age, a nadir creatinine cut-off value of 0.8 mg/dL had a predictive value with 86% sensitivity and 72% specificity (ROC curve, AUC = 0.830). Among patients diagnosed at under 1 year. of age, only two patients (6.9%) out of 29 patients with nadir creatinine values below 0.8 mg/dL had final serum creatinine values higher than the reference creatinine value. In contrast, among patients with nadir creatinine values equal to or above 0.8 mg/dL, this rate was 53.8% (14/26). This difference was statistically significant (*p* < 0.001).

The distribution of patients according to VUR status is shown in Table 2. The risk of final abnormal renal function was similar in patients without VUR (25%) and those with unilateral VUR (19.3%; *p* = 0.590). However, it was significantly higher in patients with bilateral VUR (55.2%) compared with others (Chi-square, *p* = 0.016, *p* = 0.004, respectively).

According to scintigraphy results, 13 of 31 patients with unilateral VUR had VURD. Within this group, patients with VURD had similar rates of abnormal renal function (Table 3).

**Table 3:** Unilateral VUR and renal function (RF) at last visit

		Final RF (age-specific reference values)		Total	*p*
		Normal	Abnormal		
Unilateral VUR (n = 31)	Without severe ipsilateral dysplasia (*n* = 18, 58.1%)	14	4	18	*p* = 1.000
	With severe ipsilateral dysplasia (VURD) (*n* = 13, 41.9%)	11	2	13	

*p*: Chi-square test

In the examination of additional surgical procedures among 92 patients, antireflux surgery was performed in ten patients, augmentation in eight patients, and renal transplantation in 15 patients. Nephroureterectomy was performed in nine patients, with two patients undergoing the procedure before transplantation and three patients due to ureteral augmentation.

Univariate analysis results revealed that nadir creatinine value exceeded the age-specific creatinine reference range (*p* < 0.0001), nadir creatinine value equal to or above 0.8 mg/dL (*p* < 0.0001), creatinine value at 1 year. of age exceeded the age-specific creatinine reference range (*p* < 0.007), presence of bilateral VUR (*p* = 0.003), and patients presenting with urosepsis (*p* = 0.029) were associated with final abnormal renal function. Data from patients with significant associations for final renal function in the univariate analysis were analyzed using multivariate analysis. Multivariate analysis identified nadir creatinine value exceeding the age-specific reference range as independent risk factors for final creatinine value (HR: 22.000 [2.364–204.759]; *p* = 0.007).

## Discussion

The literature has examined diagnostic and subsequent renal function to predict long-term kidney outcomes. Our study found nadir creatinine value to be significant predictors. Nadir creatinine values are widely accepted prognostic factors for predicting long-term outcomes. A review has indicated that while different cut-offs exist, a nadir creatinine value over 1 mg/dL is generally considered significant [13]. Similarly, the 1 year creatinine value has been highlighted as an important prognostic factor in predicting long-term outcomes [14]. Wu et al. [15] found that the nadir creatinine value was significant in both univariate and multivariate analyses of long-term outcomes, whereas the 1 year creatinine value was significant only in univariate analysis, similar to our study.

Concomitant VUR is clinically significant in patients with PUV. Literature indicates that concomitant VUR occurs in 30% to 80% of patients, with bilateral VUR seen in 30% of cases [16, 17]. Our data showed concomitant VUR in 68.4% of patients, with bilateral VUR in 31.5%, consistent with the literature. Univariate analysis revealed that patients with bilateral VUR had significantly higher final creatinine values than those without bilateral VUR, while no significant difference was found between those without VUR and those with unilateral VUR. However, bilateral VUR was not a significant prognostic factor in multivariate anal ysis. Literature suggests that bilateral VUR is a poor prognostic factor for long-term renal function in PUV patients [11,18]. The significant findings in our univariate analysis suggest that with an increased sample size, statistically significant results may also emerge in the multivariate analysis.

VURD, described in 1982 as a syndrome associated with high bladder pressure transfer to the refluxing kidney, thereby protecting the contralateral kidney, has since been supported by subsequent studies [19]. Subsequently, this theory supported and described pop-off mechanisms, and in 1988, it was found that long-term renal outcomes were favorable in these patients [7]. Although VURD was first described as a syndrome related to the pop-off mechanism, long-term outcomes associated with VURD remain controversial in the literature. This opposing view was first expressed in a study in 1997 [20].

There is no definitive information regarding the cut-off value that should be used when defining dysplasia. Some studies have accepted cut-offs of 10%, 15%, or 30% [21]. Based on our literature review and evaluation, we consider a 15% cut-off to be acceptable. Therefore, in this study, we defined patients with dysplasia below 15% as having severe dysplasia.

Recently published studies have not reached a clear consensus on whether VURD is a protective or worsening factor. Although some studies suggest that it is protective, it is difficult to draw firm conclusions due to the heterogeneity of the studies [22, 23]. Other studies argue that regardless of the pop-off mechanism, the presence of dysplasia may negatively impact long-term outcomes more than previously thought [24, 25], even suggesting that VURD could be a worsening factor [12].

In light of these conflicting findings, we found that unilateral VUR preserves the contralateral kidney through the pop-off mechanism, resulting in similar long-term outcomes to patients without VUR. Within the group of patients with unilateral VUR, there was no significant difference in prognosis regarding kidney function when comparing those with and without severe (< 15%) ipsilateral dysplasia. These findings indicate a protective effect of unilateral VUR, independent of the severity of kidney dysplasia. As a result, of the pop-off mechanism, we recommend that patients with dysplastic kidneys should not undergo a nephroureterectomy without a thorough evaluation.

In some patients, even after ablation, renal dysfunction may continue. This may also be related to a clinical condition called valve bladder [10]. We cannot comment on our study due to insufficient postoperative data, which is one of the limitations of our study.

The limitations of our study include its retrospective nature, also spans a long period, with some patients lost to follow-up, incomplete data for each patient, and potential variations in patient management over time. We used age-specific reference values because height and weight data, necessary for calculating GFR in pediatric patients, were insufficient in our study. Other GFR formulas designed for adults may produce inaccurate results when applied to pediatric patients. Although DMSA and MAG-3 do not show the same functions, due to the objective data, the patient’s scintigraphic study data was included in the study if available. Bladder dynamics could not be elucidated in this study due to the insufficient patients’ bladder functions, Clean Intermittent Catheterization usage status and Video Urodynamic Study data. Insufficient data regarding the degree of reflux in patients, and our inability to comment on the severity of reflux present. Although endoscopic valve ablation was primarily preferred in the applied treatments, diversion treatment was preferred for patients who were clinically worse off. In order to avoid selection bias due to this situation, the effect of the applied treatment on the final status was not included in the analysis.

## Conclusion

In summary, patients with PUV remain at risk of chronic kidney disease in the long term, despite advances in diagnosis and treatment. Lifelong follow-up is essential for these patients. In our study, patients with nadir creatinine values above the age-specific reference range were identified as being at risk of chronic kidney disease, regardless of their age at presentation. Additionally, patients with unilateral VUR had a similar prognosis to those without VUR, irrespective of dysplasia severity. Given this potential protective effect, we recommend avoiding nephrectomy without a detailed evaluation.

## Data Availability

No datasets were generated or analysed during the current study.

## Abbreviations

HUN: Hydroureteronephrosis
PUV: Posterior urethral valve
RF: Renal function
USG: Ultrasonography
VUR: Vesicoureteral reflux
VURD: Valve unilateral reflux dysplasia
